# Supplementary material for: Dimer-monomer transition defines a hyper-thermostable peptidoglycan hydrolase mined from bacterial proteome by lysin-derived antimicrobial peptide-primed screening
Source: eLife. 2024 Nov 26;13:RP98266. doi: 10.7554/eLife.98266 (PMC11594527; doi:10.7554/eLife.98266)
Supplement: Figure 2—figure supplement 1—source data 1. [file elife-98266-fig2-figsupp1-data1.zip › Figure 2-figure supplement 1-source data 1.pdf]

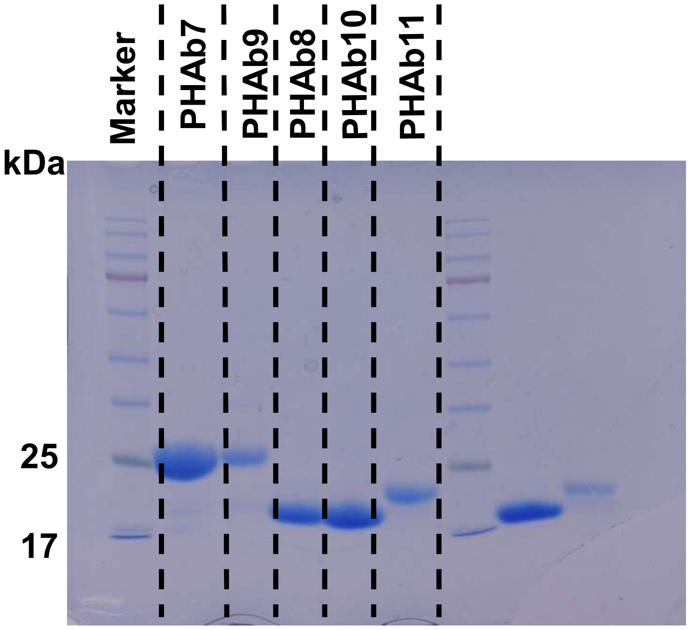

Figure 2-figure supplement 1-source data 1. PDF file containing original SDS-PAGE gel for Figure 2-figure supplement 1, indicating the relevant bands.
