# Supplementary figures and images for: Dimer-monomer transition defines a hyper-thermostable peptidoglycan hydrolase mined from bacterial proteome by lysin-derived antimicrobial peptide-primed screening
Source: eLife. 2024 Nov 26;13:RP98266. doi: 10.7554/eLife.98266 (PMC11594527; doi:10.7554/eLife.98266)

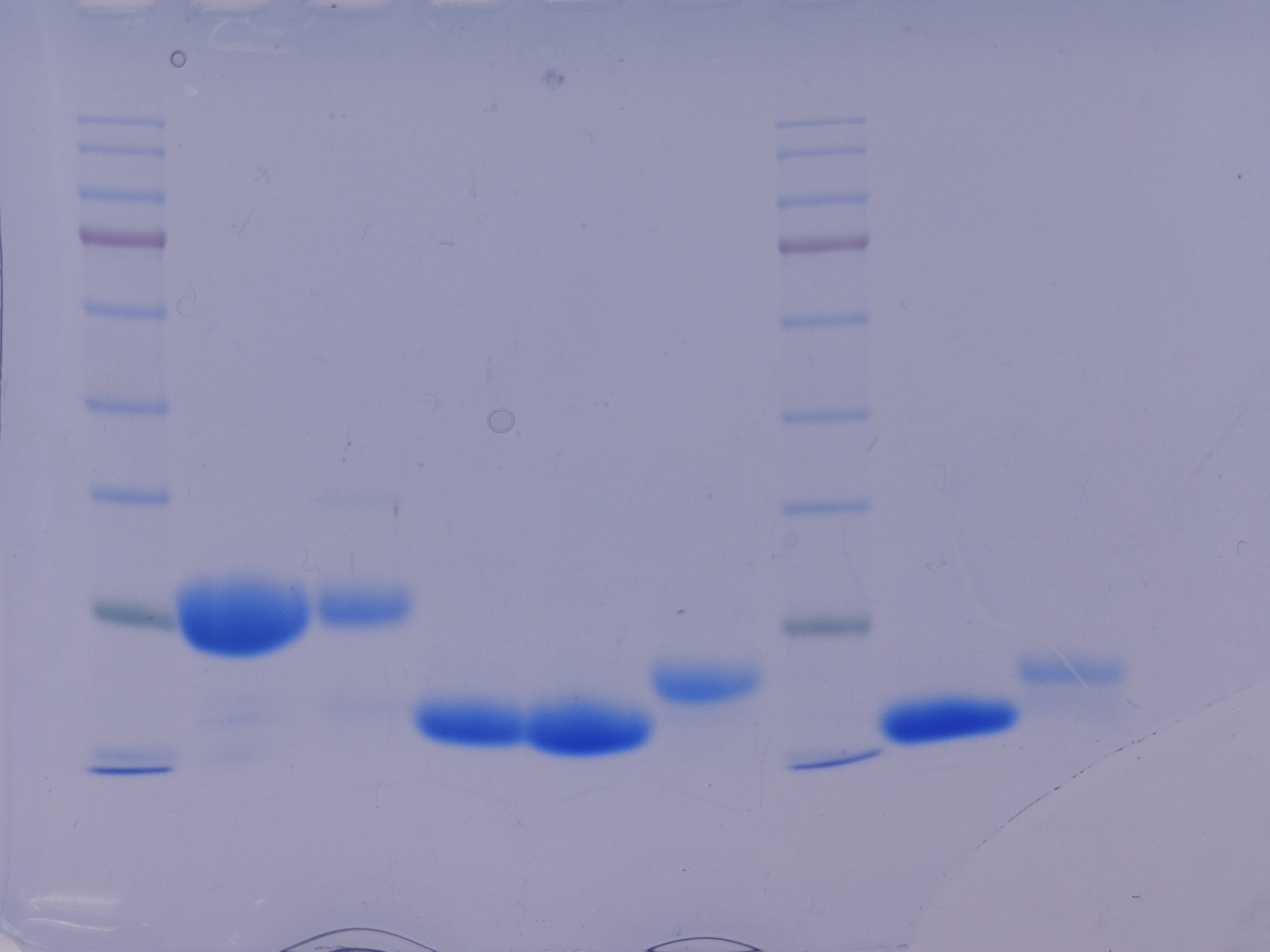

Supplement: Figure 2—figure supplement 1—source data 2. [file elife-98266-fig2-figsupp1-data2.zip › Figure 2-figure supplement 1-source data 2.jpg]

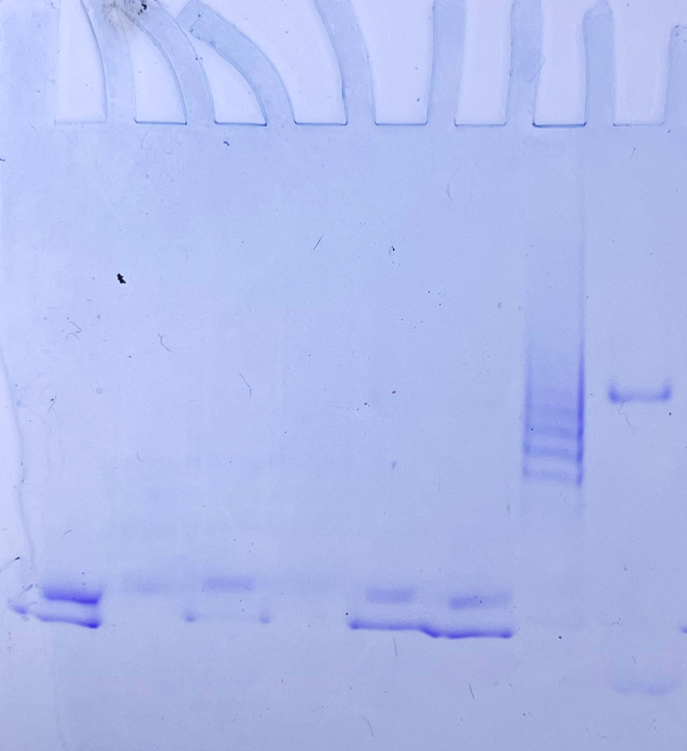

Supplement: Figure 5—source data 2. [file elife-98266-fig5-data2.zip › Figure 5-source data 2-3.tif]

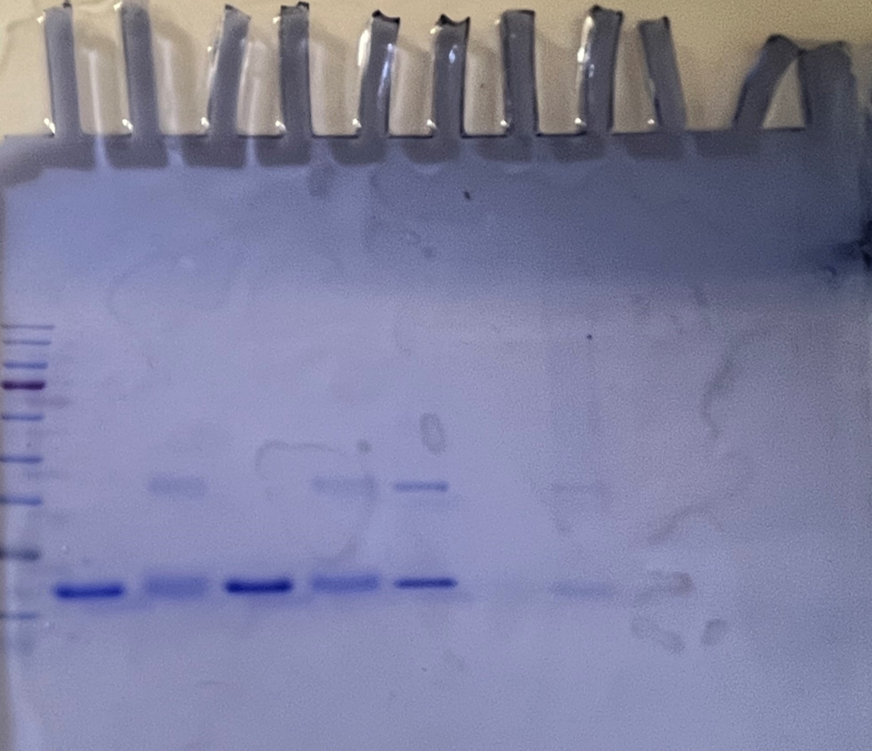

Supplement: Figure 5—source data 2. [file elife-98266-fig5-data2.zip › Figure 5-source data 2-1.tif]

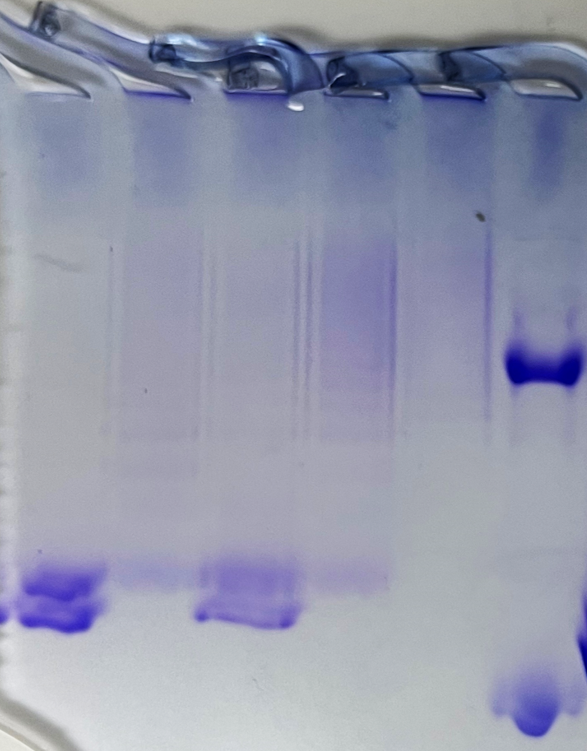

Supplement: Figure 5—source data 2. [file elife-98266-fig5-data2.zip › Figure 5-source data 2-2.tif]
