# Supplementary material for: Dimer-monomer transition defines a hyper-thermostable peptidoglycan hydrolase mined from bacterial proteome by lysin-derived antimicrobial peptide-primed screening
Source: eLife. 2024 Nov 26;13:RP98266. doi: 10.7554/eLife.98266 (PMC11594527; doi:10.7554/eLife.98266)
Supplement: Figure 5—source data 1. — The left gel corresponds to Figure 5b; the middle and the right gels correspond to Figure 5c. [file elife-98266-fig5-data1.zip › Figure 5-source data 1.pdf]

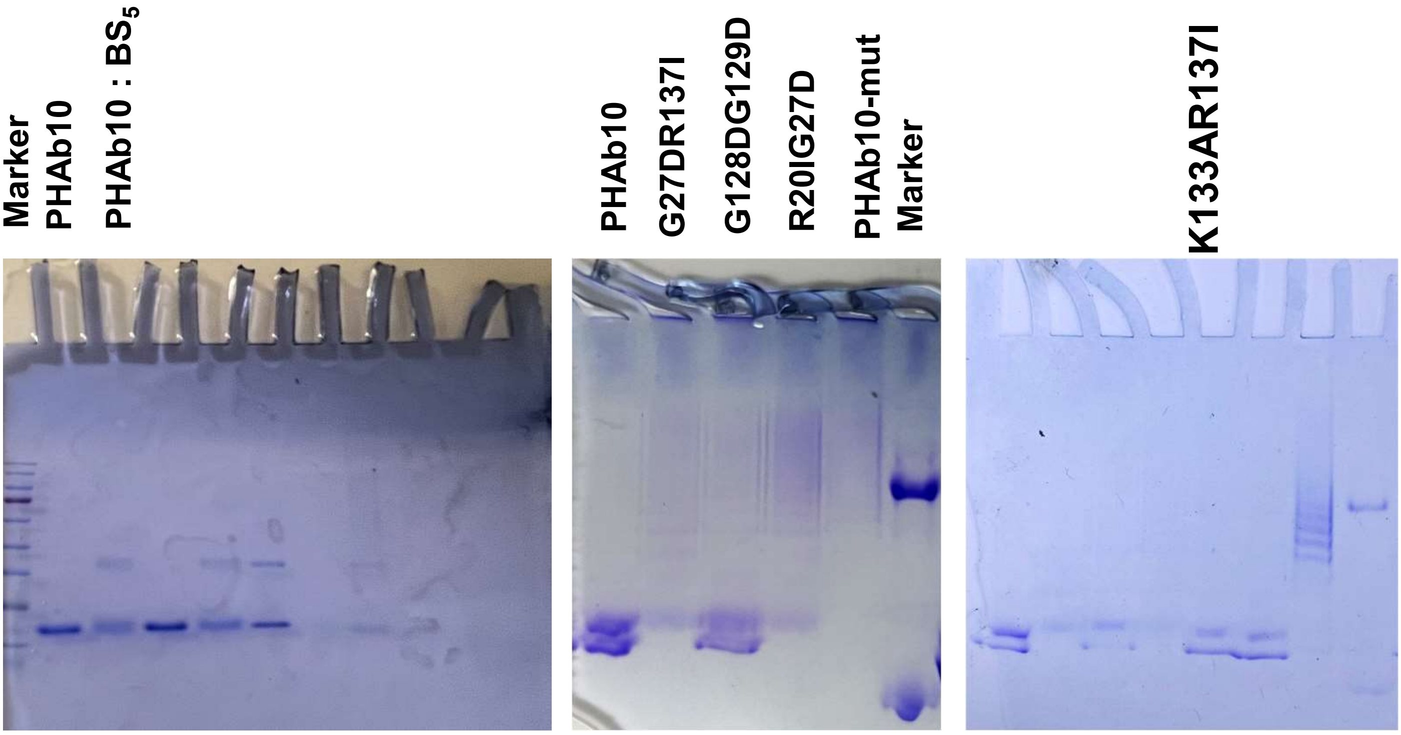

Figure 5-source data 1. PDF file containing original SDS/Native-PAGE gels for Figure 5b and 5c, indicating the relevant bands. The left gel corresponds to Figure 5b; the middle and the right gels correspond to Figure 5c.
