# Supplementary material for: Dimer-monomer transition defines a hyper-thermostable peptidoglycan hydrolase mined from bacterial proteome by lysin-derived antimicrobial peptide-primed screening
Source: eLife. 2024 Nov 26;13:RP98266. doi: 10.7554/eLife.98266 (PMC11594527; doi:10.7554/eLife.98266)
Supplement: Supplementary file 1. — (a) Physicochemical properties of putative antimicrobial peptides in PHAb10 and PHAb11. (b) Data collection and refinement statistics of PHAb8, PHAb10, and PHAb11 structures. (c) T4L-like lysozymes with characterized structures. (d) Intermolecular interactions of PHAb10 dimer. (e) Design of PHAb10 variants. (f) Bacterial strains used in this work. (g) Primers used in this study. [file elife-98266-supp1.docx]

**Supplementary Files For**

Dimer-monomer transition defines a hyper-thermostable peptidoglycan hydrolase mined from bacterial proteome by lysin-derived antimicrobial peptide-primed screening

Li Zhang^1,#^, Fen Hu^2,#^, Zirong Zhao^1,#^, Xinfeng Li^3^, Mingyue Zhong^3^, Jiajun He^1^, Fangfang Yao^4^, Xiaomei Zhang^1^, Yuxuan Mao^1^, Hongping Wei^2^, Jin He^1,^*, Hang Yang^3,5,6,^*

**Supplementary File 1a.**

**Physicochemical properties of putative antimicrobial peptides in PHAb10 and PHAb11.**

| **Parameter** | **Amino acid**  **sequence** | **Molecular weight (kDa)** | **pI** | **Negatively /positively charged residues** | **Net charge** | **Instability index** | **Aliphatic index** | **GRAVY** |
| --- | --- | --- | --- | --- | --- | --- | --- | --- |
| P10-CP | NKGDYQGAADQFLVWNKAGGKVMKGLVRRREAERALFLKK | 4550 | 10.37 | 4/10 | 6 | 39.66 | 73.25 | -0.733 |
| P11-CP | NKGDYKGAADQFLVWNKAGGKVLKGLVRRREAERALFLKK | 4532 | 10.45 | 4/11 | 7 | 35.41 | 83.00 | -0.695 |
| P11-NP | MTTKPFFDAARVIAGGKLTQAQVDELNKVVNKLAPG | 3829 | 9.52 | 3/5 | 2 | 19.74 | 89 | -0.053 |

Positively charged amino acids are labeled in red.

GRAVY: Grand average of hydropathicity.

**Supplementary File 1b.**

**Data collection and refinement statistics of PHAb8, PHAb10, and PHAb11 structures.**

| **Parameters** | **PHAb10** | **PHAb11** | **PHAb8** |
| --- | --- | --- | --- |
| **Data collection** |  |  |  |
| Wavelength (Å) | 0.9793 | 0.9793 | 0.9793 |
| Space group | *P* 1 2_1_ 1 | *P* 2 2 2_1_ | *C* 2 2 2_1_ |
| Cell dimensions |  |  |  |
| a, b, c (Å) | 31.86 73.77 78.62 | 50.01 54.9 161.31 | 79.03 156.98 47.26 |
| α, β, γ (°) | 90.00 93.02 90.00 | 90 90 90 | 90 90 90 |
| Resolution range (Å) | 31.82 - 1.52 (1.574 - 1.52) | 36.62 - 2.27 (2.351 - 2.27) | 32.06 - 2.4 (2.486 - 2.4) |
| Unique reflections | 55245 (5468) | 21246 (2049) | 11880 (1048) |
| Multiplicity | 6.8 (6.5) | 12.3 (12.4) | 12.9 (13.2) |
| Completeness (%) | 98.86 (98.17) | 99.65 (99.80) | 94.57 (88.22) |
| Mean I/sigma I | 18.36 (1.67) | 14.54 (2.02) | 10.50 (1.53) |
| Wilson B-factor (Å^2^) | 24.27 | 31.87 | 37.86 |
| R_merge_ | 0.04816 (1.097) | 0.2869 (1.486) | 0.213 (1.914) |
| R_meas_ | 0.0522 (1.192) | 0.2999 (1.55) | 0.2219 (1.991) |
| CC_1/2_ | 0.999 (0.788) | 0.992 (0.747) | 0.996 (0.579) |
| **Refinement** |  |  |  |
| Resolution (Å) | 31.82 - 1.52 | 36.62 - 2.27 | 32.06 - 2.4 |
| R_work_ | 0.1921 (0.3344) | 0.2483 (0.3092) | 0.2520 (0.3560) |
| R_free_ | 0.2151 (0.3653) | 0.2660 (0.3670) | 0.2849 (0.4396) |
| Ligands | 0 | 18 | 17 |
| Rmsd from ideal |  |  |  |
| Bond lengths (Å) | 0.004 | 0.005 | 0.010 |
| Bond angles (°) | 0.62 | 0.75 | 1.18 |
| Ramachandran plot |  |  |  |
| Favored (%) | 98.97 | 98.25 | 95.81 |
| Allowed (%) | 1.03 | 1.75 | 4.19 |
| Outliers (%) | 0.00 | 0.00 | 0.00 |
| Average B-factor | 32.18 | 37.23 | 52.84 |
| PDB Code | 7YKU | 8HDQ | 8HEM |

Statistics for the highest-resolution shell are shown in parentheses.

**Supplementary File 1c.**

**T4L-like lysozymes with characterized structures.**

| **Protein** | **Catalytic Residues** | | | **PDB ID** |
| --- | --- | --- | --- | --- |
| PHAb8 | Glu74 | Asp83 | Thr89 | 8HEM |
| PHAb10 | Glu17 | Asp26 | Thr32 | 7YKU |
| PHAb11 | Glu52 | Asp61 | Thr67 | 8HDQ |
| AcLys | Glu64 | Asp73 | Thr79 | 6ET6 |
| LysF1 | Glu15 | Asp24 | Thr30 | 7M5I |
| SpmX-Mur-*Ae* | Glu18 | Leu27 | Met33 | 6H9D |
| P22 | Glu16 | Asp25 | Thr31 | 2ANX |
| R21 | Glu35 | Asp44 | Thr50 | 3HDE |
| HEWL | Glu35 | Asp52 |  | 4HPI |
| GEWL | Glu73 |  |  | 153L |
| phage lambda lytic transglycosylase | Glu19 |  |  | 1D9U |
| T4L | Glu11 | Asp20 | Thr26 | 1LYD |

**Supplementary File 1d.**

**Intermolecular interactions of PHAb10 dimer.**

| **PHAB10 dimer** | **Serial number** | **Donor (atom 1)** | **Acceptor (atom 2)** | **Distance/Å** | **Force** |
| --- | --- | --- | --- | --- | --- |
| Head-supporting force | 1 | R20.NH1  (Chain A) | G129.O (Chain B) | 3.5 | H-bond |
|  | 2 | T104.N  (Chain B) | G27.O (Chain A) | 3.1 | H-bond |
|  | 3 | T104.OG1  (Chain B) | G27.O (Chain A) | 3.5 | H-bond |
| Midbody-supporting force | 4 | E97.N  (Chain B) | G128.O (Chain A) | 2.9 | H-bond |
|  | 5 | K67.NZ  (Chain B) | G129.O (Chain A) | 2.9 | H-bond |
| Tail-supporting force | 6 | K133.NZ  (Chain A) | D26.O (Chain B) | 2.9 | H-bond |
|  | 7 | R137.NH1  (Chain A) | G27.O (Chain B) | 2.7 | H-bond |

**Supplementary File 1e.**

**Design of PHAb10 variants.**

| **Native amino acids (Chain A)** | **Substituted amino acids** | **Mutant name** |
| --- | --- | --- |
| R20, G27 | R20I, G27D | R20I/G27D |
| G128, G129 | G128D, G129D | G128D/G129D |
| G27, R137 | G27D, R137I | G27D/R137I |
| K133, R137 | K133A, R137I | K133A/R137I |
| R20, G27, K67, G128, K133, R137 | R20E, G27T, K67W, G128N, K133A, R137N | PHAb10-mut |

**Supplementary File 1f.**

**Bacterial strains used in this work.**

| **Strain** | **Source** | **Strain** | **Source** |
| --- | --- | --- | --- |
| ***Acinetobacter baumannii*** |  | ***Klebsiella pneumoniae*** |  |
| ABA3437 | WIV^b^ | KP21 | WIV^b^ |
| CRAB2 | WIV^b^ | KP22 | WIV^b^ |
| CRAB13 | WIV^b^ | KP23 | WIV^b^ |
| CRAB36 | WIV^b^ | KP24 | WIV^b^ |
| CRAB27 | WIV^b^ | KP26 | WIV^b^ |
| CRAB28 | WIV^b^ | KP27 | WIV^b^ |
| CRAB22 | WIV^b^ | KP28 | WIV^b^ |
| CRAB26 | WIV^b^ | KP602 | WIV^b^ |
| CRAB140 | WIV^b^ | KP605 | WIV^b^ |
| CRAB143 | WIV^b^ | KP607 | WIV^b^ |
| CRAB147 | WIV^b^ | ***Pseudomonas aeruginosa*** |  |
| ***Escherichia coli*** |  | PAE2555 | WIV^b^ |
| *E. coli* 100 | WIV^b^ | PAE1 | WIV^b^ |
| *E. coli BL21* | WIV^b^ | PAE2 | WIV^b^ |
| E. coli O91 | WIV^b^ | PAE52 | WIV^b^ |
| *E. coli* O97 | WIV^b^ | PAE53 | WIV^b^ |
| *E. coli* O149 | WIV^b^ | PAE2139 | WIV^b^ |
| *E. coli* O517 | WIV^b^ | PAE2169 | WIV^b^ |
| *E. coli* MS31 | WIV^b^ | PAE2176 | WIV^b^ |
| *E. coli* MS33 | WIV^b^ | PAE2703 | WIV^b^ |
| *E. coli* MS28 | WIV^b^ | PAE3223 | WIV^b^ |
| *E. coli* MS34 | WIV^b^ | PA2-3121 | WIV^b^ |
| ***Staphylococcus aureus*** |  | PA2-3283 | WIV^b^ |
| *S. aureus* N315 | WIV^b^ | PA2-3763 | WIV^b^ |
| *S. aureus* RN4220 | WIV^b^ | PA2-3965 | WIV^b^ |
| *S. aureus* NF71 | WIV^b^ | ***Streptococcus*** |  |
| *S. aureus* 25923 | WIV^b^ | *S. pneumoniae* 17NS26 | HPPH^a^ |
| ***Enterococcus faecalis*** |  | *S. agalactiae* 12 | WIV^b^ |
| *E. faecalis* 493 | WIV^b^ | *S. agalactiae* C001 | WIV^b^ |
| *E. faecalis* 29212 | ATCC^c^ | *S. dysgalactiae* 35666 | ATCC^c^ |
| *E. faecalis* 51299 | WIV^b^ | *S. pyogenes* 12344 | ATCC^c^ |
| *E. faecalis* 571-2 | WIV^b^ | *S. mutans* 159 | WIV^b^ |
|  |  | *S. Suis* 1 | HUAHE^d^ |

a: Isolated from Henan Provincial People's Hospital, Zhengzhou, China.
b: Obtained from Wuhan Institute of Virology, Chinese Academy of Sciences, Wuhan, China.
c: Purchased from Guangdong Culture Collection Center, China.

d: Obtained from Henan University of Animal Husbandry and Economy, Zhengzhou, China.

**Supplementary File 1g.**

**Primers used in this study.**

| **Primer** | **Sequence (5’-3’)** |
| --- | --- |
| P10-Lys-F | ctttaagaaggagatataccATGggcatgagcaaaaccaccagt |
| P10-Lys-R | gtggtggtgctcgagCAGttttttcagcaggg |
| P11-N-F | agaaggagatataccATGggcatgaccaccaaaccgttttttgatgcag |
| P11-N-R | gtggtggtgctcgagCAGcagtttcagcagggtgcta |
| P11-C-F1 | aactttaagaaggagatataccatgggtggtaaaaccacc |
| P11-C-R1 | ggtggtggtgctcgagttttttcagaaacagtgcacg |
| P11-C-F2 | aactcgagcaccaccaccaccaccac |
| P11-C-R2 | ggtatatctccttcttaaagttaaacaaaattatttctagag |
| K133AR137I-F1 | tttgtttaactttaagaaggagatataccatgggcatgagcaaaaccacc |
| K133AR137I-R1 | acgacggataaccagaccggccataactttaccacc |
| K133AR137I-F2 | tggccggtctggttatccgtcgtgaagcagaac |
| K133AR137I-R2 | gctcatgcccatggtatatctccttcttaaagttaaacaaaattatttc |
| R20IG27D-F1 | accaccagtaatgcaggtctgaatctgatcaaaggttttgaaggtaaaatcctgaatgc |
| R20IG27D-R1 | gtgccaaaaccaatggtccaaacaccaacatcatcatcataggcattcaggattttacc |
| R20IG27D-F2 | ccattggttttggcaccatcaaatatccgaatggtgttcg |
| R20IG27D-R2 | cctgcattactggtggttttgctcatgcccatggtatatc |
| G128DG129D-F1 | atgagcaaaaccaccagtaatgcaggtctgaatctgatcaaag |
| G128DG129D-R1 | aactttatcatcggctttattccaaaccagaaactgatctgctgc |
| G128DG129D-F2 | aagccgatgataaagttatgaaaggtctggttcgtcgtcg |
| G128DG129D-R2 | actggtggttttgctcatgcccatggtatatctccttc |
| G27DR137I-F1 | tgatgatgttggtgtttggaccattggttttggcacc |
| G27DR137I-R1 | tctgcttcacgacggataaccagacctttcataactttaccac |
| G27DR137I-F2 | ggttatccgtcgtgaagcagaacgtgcactg |
| G27DR137I-R2 | ccaaacaccaacatcatcatcataggcattcaggcg |
